# Complex gastrointestinal vascular malformation treated with interpositional mesocaval shunt (Drapanas procedure)

**DOI:** 10.1016/j.jvscit.2023.101268

**Published:** 2023-07-16

**Authors:** Pedro J. Furtado Neves, Barbara D. Moreira, Hugo G.K. Akahane, Ricardo C.R. Moreira, Eduardo J.B. Ramos, Rafael Demarchi Malgor

**Affiliations:** a Division of Vascular and Endovascular Surgery, University of Colorado, Anschutz Medical Center, Aurora, CO; b Department of Surgery, Complexo Hospital de Clínicas, Universidade Federal do Paraná, Curitiba, PR, Brazil; c Department of Surgery, Hospital Nossa Senhora das Graças, Curitiba, PR, Brazil

Arteriovenous malformations (AVMs) remain one of the most complex diseases to treat due to their tendency to recur and their unpredictable behavior.^1^ The goal of treatment is to obliterate the nidus—the malformed tissue that communicates the high-pressure arterial system with the low-pressure venous system. Ligation of the arterial origin of the AVM is not advised because this procedure leads to the nidus promoting additional recruitment of feeding vessels from the arterial tree, often making the disease even more complex. Percutaneous embolization with small particles and glue has gained traction as treatment modalities,^2^^,^^3^ however, depending on the size of the AVM, cure is impractical. In these cases, the focus should be directed toward treating the consequences of the AVM that are most dire and requiring of focus. Gastrointestinal (GI) AVMs are frequent causes of GI bleeding, and portal AVMs can rapidly decompensate after portal vein thrombosis owing to the outflow obstruction of a high-flow AVM, leading to dilation of the venous high capacitance system, which may then continually dilate and degenerate owing to underlying portal hypertension, until GI bleeding results. We present a case of a 49-year-old man with a complex GI AVM (Fig 1) with decompensation secondary to chronic portal and superior mesenteric vein (SMV) thrombosis with recurrent lower GI bleeding, ascites, weight loss, and failure to thrive. The patient, and later the patient's surviving family members, provided consent for the publication of his case.

**Fig 1:**
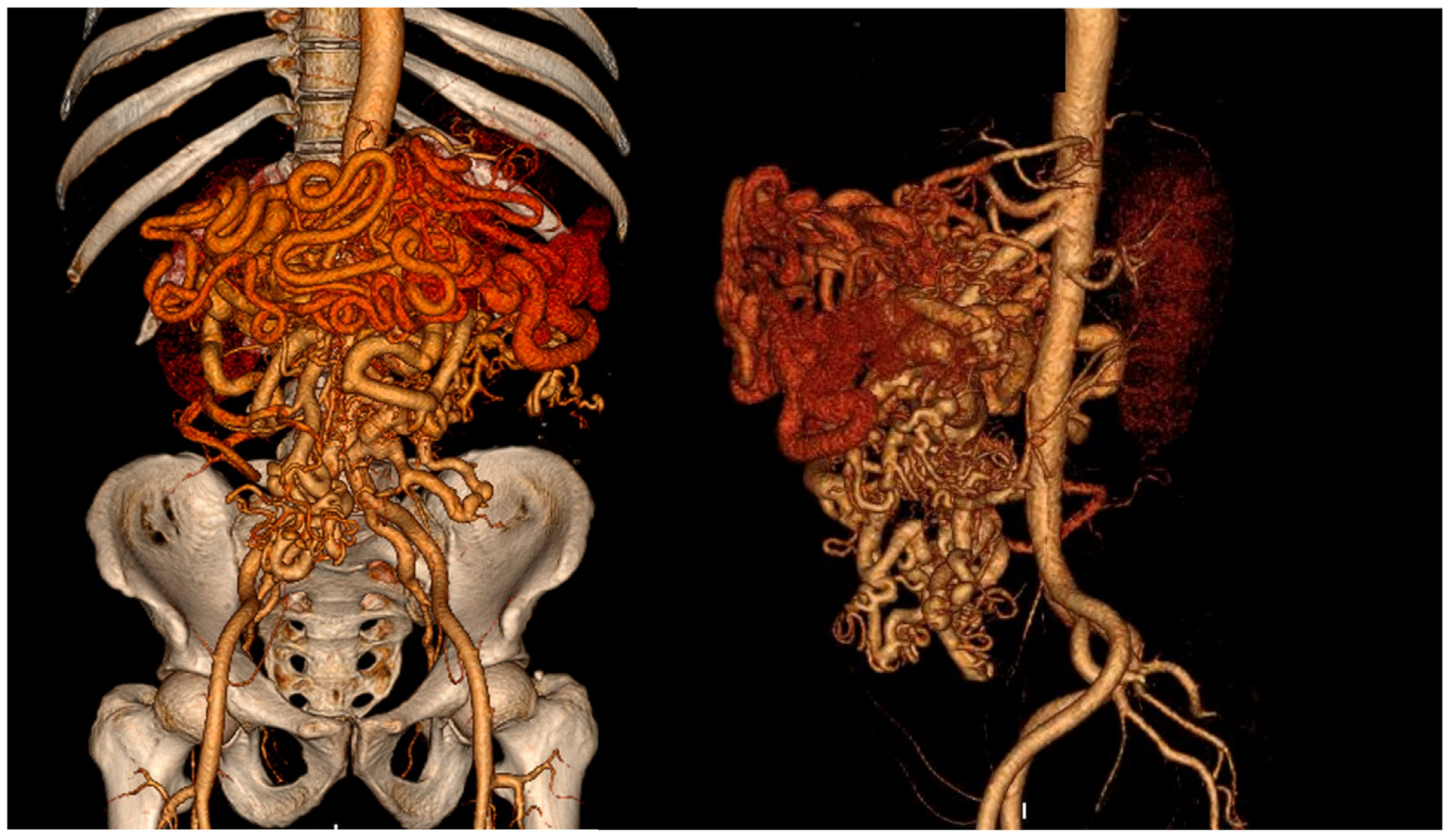
Three-dimensional multiplanar reconstruction showing the complex mesenteric arteriovenous malformation (AVM) with involvement of the celiac and superior mesenteric arteries.

The patient was a 49-year-old man with a history of portal vein and SMV thrombosis 5 years previously. He was treated with warfarin, and later switched to rivaroxaban after an episode of GI bleeding. He then progressed with persistent and worsening lower GI bleeding, as well as progressive weight loss, failure to thrive, abdominal distension, anemia, nausea, and vomiting. Anticoagulation was suspended and, despite this, GI bleeding persisted. After a workup with esophagogastroduodenoscopy and colonoscopy was unremarkable, the decision was made to investigate with a computed tomography angiography, which showed a large complex AVM with involvement of the celiac and superior mesenteric arteries (Fig 1). We proceeded with an attempt to study the AVM more clearly, as well as identify the focus of ongoing bleeding with angiography. Jejunal branches with focal bleeding were identified and embolized with particles. However, the high risk of bleeding recurrence, symptoms compatible with GI subocclusion, and significant worsening of the patient's clinical status owing to ascites, malnutrition, in the context of confirmed absence of liver cirrhosis with normal liver function tests, and liver ultrasound morphology and texture, led the multidisciplinary team in charge of patient management to discuss the possibility of open surgical treatment with the patient.

The Drapanas procedure, described by Dr Theodore Drapanas in 1972,^4^^,^^5^ consists of an interposition shunt between the SMV and the inferior vena cava and results in portal venous depressurization. The original procedure describes the use of a polyester graft, but, in this case, the team decided to harvest the superficial femoral vein, as well as performing partial enterectomy of the most afflicted bowel segment. The decision to proceed was established after lengthy discussion of the risks, benefits, and alternative therapies. Because of the patient's history of extensive portal vein and SMV thrombosis, we attributed the decompensation of the previously clinically silent GI AVM to the thrombotic event causing portal venous hypertension and progressively dilating the high capacitance venous component of the AVM. In this context, we explained to the patient that resection would treat the current GI bleeding resulting from the already dilated and fragile AVM, and the Drapanas procedure would depressurize the portal venous system and serve to prevent further dilatation of the remaining AVM and recurrence of the ascites. The main risks we discussed were the development of congestive heart failure owing to the significant increase in venous return expected after the procedure, as well as the added risk of wound healing owing to the patient's malnutrition status. However, because the malnutrition was suspected to be due to the AVM and would not resolve without intervention, the decision to proceed was made, after optimization of the patient's nutritional status with total parental nutritional support. The intraoperative aspect of the AVM is shown in Fig 2. The most affected portion of bowel was resected and sent to pathology, which confirmed bowel lumen compression, with notable mucosal sparing (Fig 3). GI transit reconstruction was performed, and the femoral vein was harvested. The shunt was then completed between a main tributary of the SMV owing to SMV occlusion and absence of recanalization and the inferior vena cava (Fig 4) and immediate thrill comparable with an arteriovenous fistula with visible pulsatile flow was noted. Revision of hemostasis and closure then ensued.

**Fig 2:**
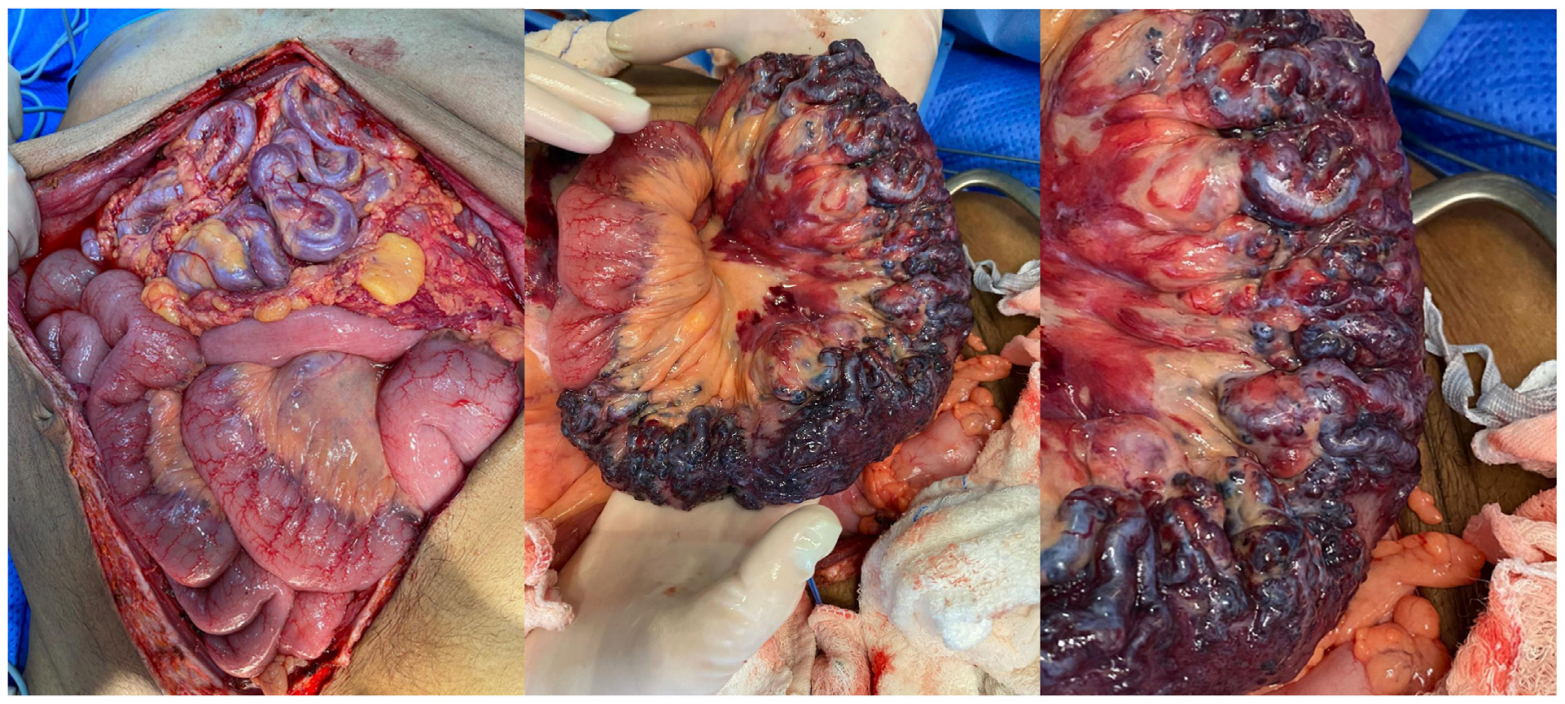
Intraoperative aspect of the complex arteriovenous malformation (AVM) and the most afflicted portion of the bowel.

**Fig 3:**
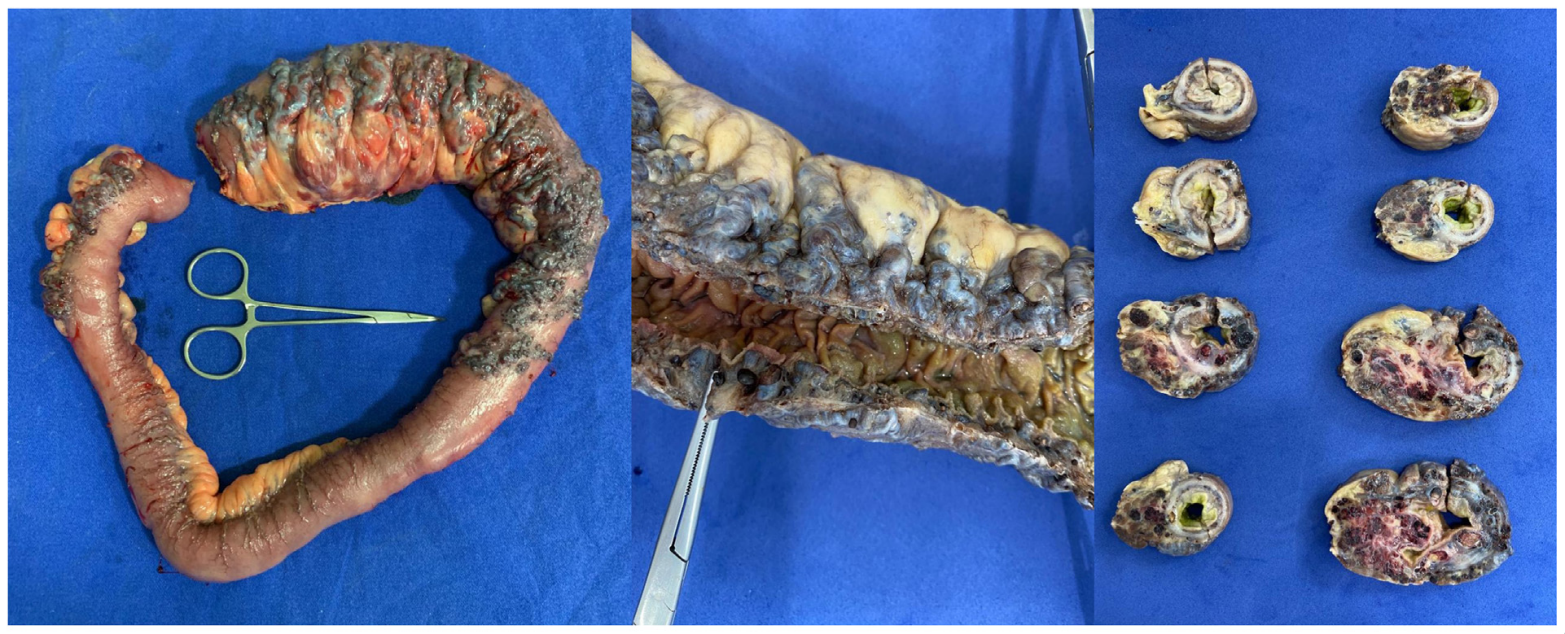
Gross macroscopy images of the resected portion of bowel. Luminal compression by the large malformation is noted. The bowel mucosa is relatively spared.

**Fig 4:**
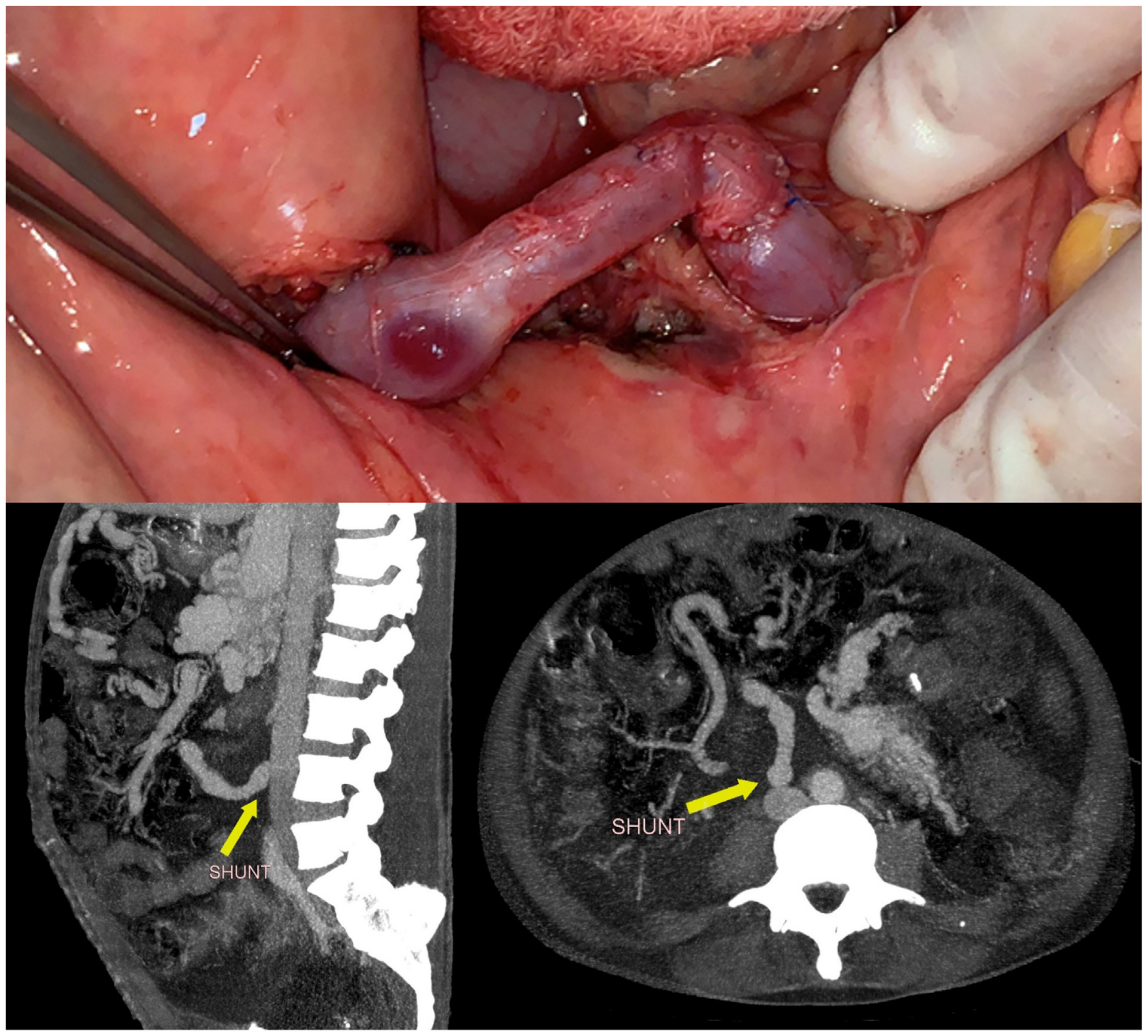
Intraoperative and postoperative follow-up imaging showing the patent mesocaval shunt using a reversed superficial femoral vein.

The patient's postoperative course was notable for prolonged postoperative ileus with 7 days of total parenteral nutrition, and the need for paracentesis with 3 L output. The patient was discharged home on postoperative day 10 on daily low-dose aspirin (100 mg). On follow-up, the shunt was noted to be patent (Fig 4), and the patient reported resolution of symptoms, weight gain, ascites nonrecurrence, and no new episodes of GI bleeding. At the 10-month follow-up, the patient was asymptomatic, and the shunt was still shown to be patent on imaging. Unfortunately, 2 weeks after this follow-up, the patient passed away from respiratory complications from coronavirus disease 2019 infection.

The elected modality of treatment was such that the resection was intended to resolve the fragile tissue responsible for the recurrent GI bleeding, and that was beyond salvage. The mesocaval shunt, in contrast, was designed as an alternative drainage of the portal circulation, to depressurize the portal system and slow or even halt the further progression of the remaining AVM, for which complete surgical resection was not possible.

In conclusion, this case demonstrates that in complex AVMs, complete resolution is often unfeasible. Therefore, focus should be directed toward treating the complications that are directly affecting the patient's quality of life and/or present a threat to life. For this, creative solutions may sometimes be required, and the assistance of experienced colleagues is invaluable. Historical procedures are not always synonymous with obsolete; therefore, a wide base of knowledge of therapeutic alternatives can make the difference in individualized patient care. Open surgical skills are still, and will always, be necessary, and the endeavor of excellence in this regard must be actively pursued, lest they face neglect.

## Appendix (online only)

Supplementary Video (online only) Video detailing the decision-making process, the surgical procedure and follow-up.
